# Ectodysplasin-A (EDA) Signaling Cross-Talk in Skeletogenesis

**DOI:** 10.1007/s00223-026-01502-0

**Published:** 2026-03-09

**Authors:** Ehsan Pashay Ahi, Jacqueline Moustakas-Verho, Pooja Singh

**Affiliations:** 1 Organismal and Evolutionary Biology Research Programme, Faculty of Biological and Environmental Sciences, University of Helsinki, Viikinkaari 9, 00014 Helsinki, Finland; 2 Natural Resources Institute Finland (Luke), Helsinki, Finland; 3 Division of Aquatic Ecology and Evolution, Institute of Ecology and Evolution, University of Bern, Bern, Switzerland; 4 Department of Fish Ecology and Evolution, Swiss Federal Institute of Aquatic Science and Technology (EAWAG), Kastanienbaum, Switzerland

## Abstract

Skeletal morphogenesis is a highly complicated interaction cascade of molecular cues, with the Ectodysplasin-A (EDA) pathway emerging as a potentially important contributor to this biological process. This review focuses on the molecular complexity of the EDA pathway’s role in shaping the diverse skeletal architectures observed in vertebrate models studied to date, particularly in fish and mammals. At the molecular level, we first discuss the signaling cascades initiated by EDA and briefly explore its impact on skeletal development. Insights into the transcriptional regulation and downstream effectors activated by EDA provide a greater understanding of its influence on skeletal formation. Beyond its standalone role in skeletogenesis, the review mainly focuses on the dynamic cross-talk between the EDA pathway and other important skeletogenic/morphogenic pathways. The multi-layered interplay with signaling networks, such as BMP, Hedgehog, Wnt, and FGF, highlights the integration of this pathway into broader molecular process governing skeletal morphogenesis. The physiological role of EDA in skeletal tissues appears highly context-dependent, varying with the interacting pathway, cell type, and developmental stage. We explore instances where EDA acts as a conductor, harmonizing its effects with those of other pathways to achieve distinct outcomes in skeletal diversity, and propose a conceptual framework in which EDA integrates these inputs through shared transcriptional hubs, notably NF-κB and NFATc1, in a tissue- and stage-specific manner. By summarizing the interactions of EDA and their associated physiological roles, we provide a comprehensive perspective on the EDA-dependent molecular underpinnings of skeletal diversity, offering new and valuable insights for future research and potential applications in skeletal biology.

## Introduction

Skeletogenesis, the formation of bones and other skeletal structures in animals, is a highly complicated process that involves a multitude of molecular players. One such emerging contributor is Ectodysplasin (EDA), a molecule known to be important for shaping the outer layers of an organism’s body [1–3]. Dysfunctions in the EDA gene can lead to X-linked hypohidrotic ectodermal dysplasia (XLHED), a condition characterized by missing teeth, sparse hair, and sweat gland problems [4–6]. Studies on animal models, such as Tabby mice with EDA mutations, as well as EDA-deficient fish models, have revealed abnormalities in bone shape and density, particularly in the facial region and appendages, prompting further investigation into EDA’s role in craniofacial and dermal skeletogenesis [7, 8]. Despite some understanding of EDA’s function, many aspects of its involvement in bone and cartilage development remain elusive, including the extent of its direct effects on osteoblasts and chondrocytes, the context-dependence of its cross-talk with other skeletogenic pathways, and the degree to which these mechanisms are conserved or divergent across vertebrate lineages, highlighting the need for deeper exploration. Hence, we believe that the time has come to gather all the relevant recent findings about the EDA pathway and its molecular interactions with other known skeletogenic pathways in both dermal and endochondral skeletal contexts to provide a comprehensive picture of how integrating EDA signaling in skeletogenesis can guide future investigations in the field of skeletal biology. In this review, we focus on the complex molecular mechanisms underlying the role of EDA in skeletogenesis, with a specific focus on fish and mammals. Firstly, we highlight the signaling pathways and molecular processes through which EDA influences bone and cartilage formation. Secondly, we explore various crosstalk between the EDA pathway and other skeletogenic signaling pathways, such as BMP, Hedgehog, Wnt, and FGF pathways, that are well-known to affect skeletal development and morphogenesis across vertebrates [6, 9–11], as well as growth factor, nuclear receptor and microenvironment-responsive pathways for which the evidence is currently more fragmentary but highly suggestive. For these latter interactions, we specifically distinguish between experimentally demonstrated links and more speculative models inferred from shared downstream effectors such as NF-κB and NFATc1, and we present the latter primarily as hypotheses to be tested.

In addition, throughout the article, we distinguish between dermal skeletal elements in teleosts (for example, scales and fin rays) and predominantly endochondral bones in mammals, and we explicitly indicate when EDA-related mechanisms are specific to one skeletal compartment or taxon. We discussed fish models as evolutionary and mechanistic complements to mammalian data where appropriate, rather than assuming that mechanisms identified in one lineage necessarily apply to all vertebrates. The overarching idea is to better understand how these pathways collectively regulate skeletal development and how EDA may act as an integrator of their inputs. Through this pathway-based framework of molecular interactions, we discuss empirical studies that have investigated the influence of EDA on skeletal development, morphogenesis, regeneration, and adaptation. In summary, this review endeavors to provide a timely and comprehensive understanding of the role of EDA in shaping skeletal structures in animals, and to articulate a conceptual framework in which EDA functions as a context-dependent “conductor” that coordinates multiple signaling cascades during skeletogenesis. Our synthesis will contribute valuable insights to the fields of developmental, evolutionary, and regenerative biology, with potential implications for the development of novel therapeutic strategies targeting skeletal-related disorders, and will formulate specific mechanistic questions and hypotheses to guide future experimental work on EDA signaling in skeletal tissues.

## Role of the EDA Pathway in Skeletal Formation

*Basic characteristics* Ectodysplasin-A (EDA) is a type II transmembrane protein that belongs to the tumor necrosis factor (TNF) ligand superfamily and plays a central role in the development of ectodermal derivatives. It is synthesized as a membrane-bound precursor that undergoes proteolytic cleavage by furin-like convertases, releasing a soluble, biologically active form [4, 12]. The cleavage occurs at a conserved furin recognition site (R-X-K/R-R), which is essential for generating the soluble trimeric ligand. Mutations that disrupt this cleavage site have been shown to impair EDA secretion and reduce signaling activity [13, 14].

The mature EDA protein contains a conserved C-terminal TNF homology domain (THD), which facilitates trimerization and receptor binding [15]. As with other TNF superfamily ligands, EDA forms stable homotrimers, a configuration required for effective receptor clustering and downstream signaling [15, 16]. This trimerization is mediated by the THD and has been confirmed through crystallographic and cryo-EM structural analyses [17]. Alternative splicing of the *EDA* gene (on chromosome Xq12-q13.1 in humans) gives rise to at least two major isoforms in humans; *EDA-A1* and *EDA-A2*, which differ by the presence of a short amino acid insertion [2, 4]. This difference confers selective binding to distinct receptors: EDA-A1 binds exclusively to the EDA receptor (EDAR), while EDA-A2 interacts with the X-linked ectodysplasin-A2 receptor (XEDAR).

Ligand-receptor engagement activates downstream signaling cascades, most notably the nuclear factor kappa B (NF-κB) pathway, through adaptor proteins such as EDARADD and TRAF6 [18–20]. Signal initiation depends on ligand-induced receptor oligomerization, which facilitates the recruitment of EDARADD via death domain (DD) interactions [21]. These DD: DD interactions rely on structurally conserved hydrophobic surfaces and salt bridges, forming a stable signaling complex that recruits TRAF6 and propagates NF-κB activation. Mutations in the death domain of EDAR or EDARADD can disrupt complex formation and abolish downstream signaling [21].

Beyond the THD, EDA also contains extracellular and intracellular motifs that regulate its proteolytic processing, membrane trafficking, and stability [12, 22]. The protein undergoes N-linked glycosylation, which is important for proper folding and secretion. There is also some evidence that EDA can associate with cell surface heparan sulfate proteoglycans, potentially modulating its local concentration and spatial distribution in the extracellular matrix [23]. Furthermore, structural modeling has suggested pH-sensitive conformational flexibility in the soluble form of EDA, which may influence receptor binding dynamics in specific tissue microenvironments [23, 24].

These biochemical features support the view of EDA as an important morphogen with tightly regulated post-translational processing, isoform-specific receptor affinity, and signal transduction capacity, all of which contribute to its spatial and temporal specificity during vertebrate development [25]. Importantly, the furin cleavage site, THD trimerization interface, and key receptor-interaction residues are highly conserved across vertebrate species [26], reflecting strong evolutionary constraints on the structural elements essential for EDA function.

*Skeletal cell differentiation* Mutations in the Eda gene lead to X-linked hypohidrotic ectodermal dysplasia (XLHED), characterized by abnormalities in ectodermal appendages and sometimes mesodermal features like craniofacial dysmorphism [8]. Study of Eda1-deficient mice has suggested an important role for EDA signaling on bone development through affecting both osteoblast and osteoclast differentiation, as it is activity was found to be required in Osterix (Osx)+ osteoblasts and Edar-positive osteoclasts in this mammalian model [27, 28]. The Eda1-deficient mice exhibited osteopetrosis-like changes with reduced marrow space and mature osteoclastic differentiation, as well as impaired osteoclast function, both indicating altered bone homeostasis [27]. The same study showed that EDA treatment restores these effects in osteoclasts (through Nfatc1 translocation and NF-κB activity), indicating its potential therapeutic implications [27]. Early postnatal EDA treatment in Eda1-deficient mice normalized vertebral bone density in adults, indicating the requirement of EDA1 for normal osteogenesis in later life stages as well [28]. A similar finding about the osteogenic activity of EDA signaling (through EDA treatment) has been already reported in study of osteosarcoma in human cells [29]. Compared to bone, less is known about the involvement of EDA signaling in cartilage development and morphogenesis; however, initial studies have found roles for the EDA pathway in the chondrogenic activities of fish and mammals [7, 30–33]. In zebrafish, for instance, research has demonstrated that EDA signaling regulates the differentiation of skeletal progenitor cells into chondrocytes, a key process in cartilage development [31, 34, 35]. Activation of EDA signaling has been shown to promote chondrocyte proliferation and extracellular matrix synthesis, which are essential for cartilage formation and morphogenesis [31]. Similarly, studies in postnatal mice have highlighted the importance of EDA signaling in chondrogenesis and cartilage development, suggesting that some EDA-dependent chondrogenic functions may be conserved, even though the cellular contexts and skeletal elements differ between fish and mammals. EDA signaling has been correlated with the expression of genes associated with chondrocyte differentiation, suggesting a potential influence on cartilage formation and growth [7]. Moreover, clinical studies have identified mutations in genes encoding components of the EDA pathway in patients with skeletal dysplasias, further implicating the pathway in cartilage-related disorders [33].

*Skeletal regeneration* The ectodysplasin A (Eda) pathway appears to play a role in skeletal tissue regeneration in several vertebrate models studied so far, most notably zebrafish and other teleosts, as well as mice and humans. Emerging evidence in fish and mammalian models, has begun to elucidate the molecular mechanisms underlying Eda-mediated osteogenesis and chondrogenesis, but these mechanisms have only been directly characterized in a limited number of systems. For instance, it has been demonstrated that Eda signaling regulates the differentiation of skeletal progenitor cells into osteoblasts and chondrocytes through activation of the NF-κB pathway [31]. Furthermore, investigations in mammalian models, including mice and humans, have provided valuable insights into the role of the Eda pathway in skeletal tissue regeneration [9]. This study showed that Eda signaling controls the expression of genes involved in osteoblast and chondrocyte differentiation in mice, while clinical studies identified mutations in genes encoding Eda pathway components in patients with skeletal dysplasias with impaired regenerative capacities in these tissues [9, 36, 37]. Moreover, recent studies have highlighted the therapeutic potential of targeting the Eda pathway for bone and cartilage regeneration [7]. It is demonstrated that activation of Eda signaling promotes bone regeneration by enhancing the proliferation and differentiation of osteoblasts in mouse models. Similarly, it has been shown that Eda signaling plays roles in cartilage repair and Eda treatment significantly improved cartilage regeneration by enhancing chondrocyte proliferation and extracellular matrix synthesis [7, 30]. Furthermore, molecular studies have revealed the multi-layered regulatory network of the Eda pathway in skeletal tissue regeneration. Overall, these findings reveal the necessity of further research on role of the Eda pathway in skeletal tissue regeneration and highlight its therapeutic potential for treating bone and cartilage-related disorders and injuries.

*Dermal skeletogenesis* The dermal skeleton, comprising the external morphology of adult fish, encompasses various elements such as the skull’s dermocranium, opercular lateral bones, scales, fin rays, teeth, and gill rakers. Unlike the endochondral ossification process in which osteoblasts deposit organic matrix over a chondrogenic scaffold, dermal skeletal elements arise from direct mineralization of a collagenous matrix deposited by dermal fibroblasts, closely associated with the epidermis [38]. Development and patterning of dermal elements are akin to epidermal appendages and are regulated by reciprocal signaling between epithelium and mesenchyme [39]. Most dermal skeletal elements in teleosts do not form during larval development but rather through juvenile metamorphosis, with variations playing a significant role in fish population adaptations to diverse environments, and mutations in genes like ectodysplasin (eda) affecting these elements, suggesting an ancient and conserved role of Eda signaling in dermal skeleton formation and patterning in teleosts, and possibly more broadly in vertebrate dermal structures [31]. Throughout vertebrate evolution, from fish to tetrapods, there has been a transition in dermal structures, with lateral bones, scales, dermal plates, and fin rays either reduced or lost, accompanied by the evolution of specialized keratinized integumentary appendages [40]. The diversity of form in extant bony fishes involves modifications in the size, shape, and number of scales, fin rays, cranial dermal bones, and teeth. Mutations in genes like ectodysplasin (eda) and edar affecting the Eda signaling pathway, crucial for hair and teeth formation in mammals, reveal the importance of this pathway in fish skeletal morphogenesis [9]. Loss of Eda signaling in zebrafish mirrors human hereditary disease hypohidrotic ectodermal dysplasia (HED) phenotypes, highlighting zebrafish mutants as genetic models of this disease [9, 31]. The activation of Eda signaling in fish epidermis contributes to the formation of an epidermal placode, resembling early development in other vertebrate integumentary appendages. Variations in the expressivity of dominant alleles sensitive to background modifiers and organ-specific responses to reduction of Eda signaling suggest these alleles as potential drivers of morphological variation in evolution [31, 41–43]. Furthermore, other major pathway such as Wnt signaling can act as an upstream regulator of EDA during the morphogenesis of dermal bones such as scales and armor plates [41, 43]. It is important to note, however, that these EDA-mediated mechanisms in teleost dermal skeletal elements operate in a developmental framework that is distinct from mammalian endochondral bones, and should therefore be interpreted primarily as evolutionarily informative analogues rather than as direct mechanistic models for all aspects of mammalian osteogenesis.

## Cross-Talk Between EDA and Major Skeletogenic Pathways

Across the pathways discussed below, many of the EDA-related mechanisms appear to be conserved at the level of core components (for example, the use of NF-κB and NFATc1 as shared downstream hubs and the involvement of Wnt, BMP or Hedgehog ligands), yet their deployment and phenotypic consequences differ markedly between teleost dermal elements and mammalian endochondral bones. In fish, most experimental evidence links EDA to the patterning and growth of epidermis-associated skeletal structures such as scales and fin rays, whereas in mammals EDA often acts more indirectly on osteoblasts, osteoclasts and chondrocytes through pathway cross-talk and transcriptional integration. In the following sections, which provide the main pathway-based synthesis combining data from fish and mammals, we therefore use this comparative framework to emphasize not only parallels but also points of divergence in EDA function, signaling interactions and skeletal phenotypes, in order to clarify which aspects are likely to represent conserved ancestral roles and which may reflect lineage- or tissue-specific specializations. Because direct comparative studies of EDA-dependent pathway cross-talk across fish and mammals are still limited for many of the signaling systems discussed, we do not attempt an exhaustive pathway-by-pathway analysis of conservation versus divergence; instead, this paragraph serves as an explicit caveat and guide for the reader in interpreting the evidence summarized below.

### Transforming Growth Factor-beta Signaling Pathways

The TGF-β superfamily, a group of structurally related polypeptides conserved across the animal kingdom, includes members synthesized as large precursors that undergo proteolytic cleavage, releasing mature and active forms (e.g., BMPs) or mature and latent forms (e.g., TGF-β) [44]. Secreted TGF-βs bind to transmembrane receptors, regulated by various factors and transmit signals through intracellular SMAD proteins, regulating target genes and influencing biological processes, including ECM synthesis and skeletal remodeling [44–46]. TGF-β subfamily members and their receptors contribute to the development and morphogenesis of various skeletal structures [11, 47, 48]. TGF-β1, a ubiquitously expressed member, plays a key role in skeletogenesis, influencing skeletal metabolism and the balance between bone formation and resorption [49, 50]. TGF-β pathways’ effects on skeletal morphogenesis involve modulation of extracellular matrix (ECM) production, as well as regulation of major skeletogenic factors including BMPs, Runx2, RANK, OPG, and twist1 [47, 49, 51, 52].

In mammals, it has been shown that EDA signal can act upstream of several components of TGF-β pathway during embryonic development, for example, in the regulation of the expression of *Bambi*, a pseudoreceptor related to the Tgf-β superfamily type I receptors, thereby inhibiting the activity of Tgf-β signaling [30]. Another layer of potential cross-talk between EDA and TGF-β pathways can be through regulation of the Smad7 transcription factor by EDA activation [30, 53]. Inhibitory cross-talk between EDA and TGF-β pathways has also been reported during tooth development; however, the detailed molecular mediators of these interactions have remained unexplored [54]. In fish, regulatory interactions between components of EDA and TGF-β pathways have been predicted during dermal bone (scale) regeneration in zebrafish and related teleosts [34], whereas in mammals the better-established interplay between EDA and TGF-β so far concerns craniofacial and tooth epithelia in the mouse and human orofacial region rather than dermal bone. During craniofacial skeletal development in both mammals and fish, EDA through its downstream effector, NF-KB, regulates palatal morphogenesis and TGF-β signal is also an essential player in this process, acting upstream of NF-KB [55]. This may indicate the competitive interactions between the two signals in regulating shared downstream effectors during skeletogenesis. At present, the predicted EDA–TGF-β interactions in teleost scale regeneration are therefore best interpreted as evolutionary parallels to the better established interplay between these pathways in mammalian craniofacial tissues, rather than as direct models for endochondral bone. Overall, the physiological output of EDA and TGF-β interaction in skeletal tissues appears to be involved in developmental growth mechanisms.

### Bone Morphogenetic Protein Signaling Pathways

Bone morphogenetic proteins (BMPs) are part of the TGF-β superfamily and signal through specific BMP receptors (BMPRs), activating SMAD proteins [56, 57]. Signaling modulation occurs through extracellular and intracellular BMP antagonists, differential SMAD regulation, inhibitors, and negative feedback loops [56, 58]. In vertebrates, BMPs contribute to diverse skeletal structures; Bmp4 signaling, for instance, influences tooth and neural crest-derived skeletal development [59, 60]. During early vertebrate development, ectodermal BMP signals interact with other morphogens to establish gene expression domains [61]. BMP signaling regulates musculoskeletal cell differentiation and chondrogenesis. Inhibiting BMP signaling affects bone and cartilage formation differently across developmental stages, with high BMP levels promoting chondrogenesis over osteogenesis. BMP signals regulate numerous TFs, including essential skeletogenic factors, orchestrating gene expression networks in a dose-dependent manner [62].

**Fig. 1:**
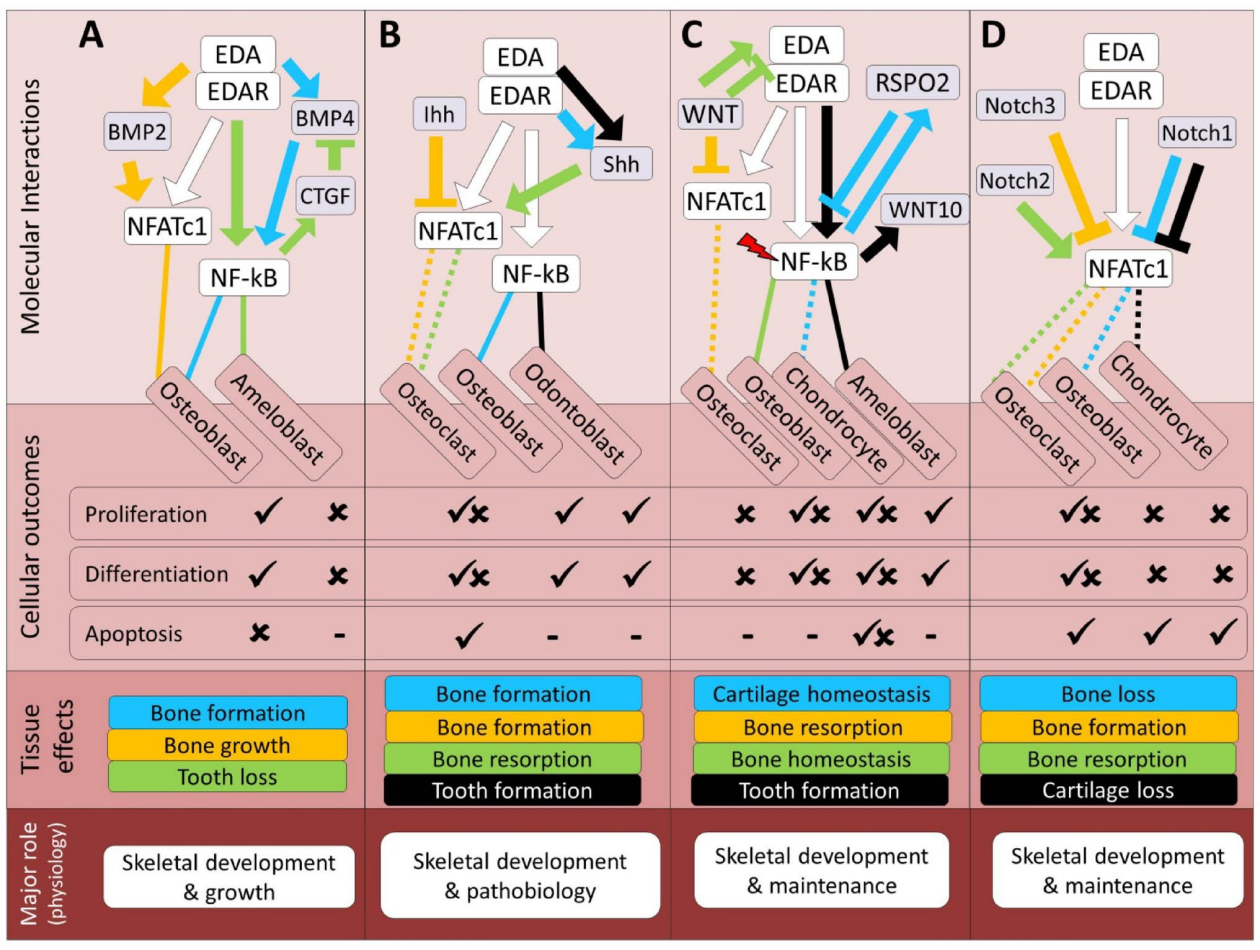
Predicted regulatory crosstalk of the EDA pathway components with major skeletal development pathways. (**A–D**) Molecular and cellular details of EDA signaling crosstalk with the BMP, Hedgehog, Notch, and Wnt pathways, respectively, along with their associated skeletal outcomes at the tissue and physiological levels. In the molecular interactions section, arrows and block heads represent regulatory induction and inhibition, respectively. The color of lines connecting EDA signal to each cell type follows the color of the specified interaction, and dashed and solid lines respectively represent indirect and direct regulatory connections between the upstream components of EDA and other pathways. In addition, red slash symbols above certain genes indicate upstream components that detect mechanical signals within each molecular signaling. The color coding of skeletal effects corresponds to the colors of related regulatory interactions in the molecular section. In the cellular section, ticks and crosses denote the promotion and impairment of processes, based on the crosstalk studied. The lowest section presents the primary expected physiological role of EDA in the context of its crosstalk with each pathway

The regulatory crosstalk between the EDA/EDAR and BMP pathways has been implicated in skeletogenesis and the development of skeletal tissues. Research indicates that during fin formation in medaka, the activation of the EDA/EDAR signaling is necessary for osteoblast differentiation and typically precedes the expression of BMP2b, suggesting a sequential interplay during fin skeletogenesis [63]. This EDA–BMP interaction in the fin appears to involve only the dermal (but not endochondral) bone structures; namely, the fin rays [63]. This pattern is echoed in zebrafish, where EDA/EDAR signaling is also essential for BMP2b expression and subsequent dermal bone formation during scale and fin development [31]. These teleost findings are best viewed as evolutionary parallels that illustrate how EDA–BMP coordination can shape dermal skeletal elements, whereas in mammals the available data point to a more indirect relationship in which BMP pathways modulate EDA-related transcriptional regulators such as *Nfatc1*. Moreover, EDA-A2 has been implicated in bone formation, possibly through a synergistic interaction with BMP-4, which together activate caspase-3 mediated osteoblast differentiation [29]. In endochondral-derived bone tissue, BMP pathways regulate major transcription factors involved in the EDA pathway, such as Nfatc1, where BMP-2 enhances osteoblast proliferation and differentiation by inducing Nfatc1 expression through SMAD1/5 binding to its promoter [64]. Moreover, during tooth development, EDA-A1 is known to inhibit BMP4 expression via NF-kB-dependent induction of Ccn2/CTGF, a BMP inhibitor, thus inhibiting ameloblast differentiation and tooth formation [30, 65]. These studies collectively point to a potential complex interplay between EDA/EDAR and BMP signaling in regulating various aspects of skeletal development (summarized in Fig. 1A). Thus, the physiological output of EDA and BMP interactions in skeletal tissues appears, based on current evidence, to be particularly relevant for developmental growth and patterning.

### Hedgehog Signaling Pathway

One of the earliest basic genetic pathways for animal development to be discovered was hedgehog (Hh) signaling, which has been thoroughly investigated in a number of model species [66]. Desert hedgehog (dhh), Sonic hedgehog (shh), and Indian hedgehog (ihh) are the three main kinds of Hh proteins that are encoded by genes in vertebrates that follow whole genome duplication (WGD) and functional diversification. The transmembrane protein called Dispatched in the generating cells releases the activated forms of Hh proteins, which are then bound by Ptch1 and Ptch2 receptors on target cells that are sensitive. Upon binding Hh, Ptch releases Smoothened (Smo), an additional membrane protein that links with the Gli family of transcription factors to control target gene transcription [67]. During the development of skeletal structures and as an early initiator of cartilage cell differentiation, the functions of Hh signaling components have been intensively researched [68, 69]. Shh plays a critical role as an intermediary in the formation of skeletal structures. Shh derived from the endoderm assures the continued existence of neural crest cells in the craniofacial skeleton [70]. Ihh, an additional Hh ligand that plays a variety of functions in skeletogenesis, modulates the palatogenesis process, and promotes chondrocyte differentiation and proliferation, osteoblastogenesis, and ossification [69, 71, 72].

The regulatory crosstalk between the Hh and EDA pathways has been shown to contribute to skeletal development and skeletogenesis in the vertebrate models studied to date, particularly in teleost fish and mice. In zebrafish and medaka, EDA/EDAR signaling notably influences the expression of Sonic hedgehog (shh), which is essential for the anterior-posterior patterning and bone formation in paired fins [31, 63]. Specifically, mutations in the *eda* gene can lead to a total absence of *shh* expression, inhibiting osteoblast proliferation and differentiation, and consequently impairing fin formation and regeneration [63]. Similarly, in zebrafish scales and cichlid fish, EDA-mediated activation of shh is necessary for bone formation and the differentiation of osteoblast-like cells [43, 73], with variations in *shh* expression suggested as a mechanism for adaptive morphological divergence [43, 74]. In cichlids, this signaling synergy extends to the development of jaw cartilage [75]. These teleost examples thus represent functional analogies to Eda–Shh interactions in mammals, where in mice, during early tooth development, Shh is induced by Eda signaling, promoting the growth of the dental bud [2, 30], even though comparable roles in mammalian endochondral long bones have not yet been demonstrated. Moreover, in pathological contexts, such as jaw bone diseases, Shh can drive *Nfatc1* expression and activity, promoting osteoclast proliferation and differentiation, which leads to bone resorption [76], while Indian hedgehog (Ihh) activation in endochondral bone tissue (trabecular and cortical bone) can inhibit *Nfatc1* expression and osteoclastogenesis, favoring osteoblast differentiation and bone formation [77]. This evidence highlights a complex interplay between EDA and Hh pathways, highlighting their potential indirect interactions through transcriptional regulation mechanisms that significantly impact bone and cartilage development and regeneration (Fig. 1B). Thus, at the physiological level, EDA-Hh interactions might not only play a role during skeletal development and patterning but also under pathological conditions in these tissues.

### Wnt/β-Catenin Signaling Pathway

Wnts, which are a family of secreted glycoproteins, are essential for key processes such as embryonic growth and morphological development, as they activate numerous pathways for signal transduction. Due to their critical role in skeletogenesis, potential for therapeutic skeletal regeneration and master modulatory role via links with multiple morphogenic channels [78, 79]. Modest variations in the strength, periodicity, and duration of Wnt signals influence developmental and adaptive skeletogenesis, bone remodeling, and regeneration [78, 80, 81]. Bone mineral density is correlated with polymorphisms in Wnt pathway components. Within the canonical pathway, Wnts establish a binding interaction with a transmembrane receptor of the Frizzled (FZD) family in its extracellular domain. Facilitating the binding process are co-receptors including LRPs (LRP5/6). A sequence of molecular events is initiated by the Wnt/LRPs/FZD complex, culminating in the establishment of a gene regulatory complex in the nucleus between β-catenin and additional factors (Lef and TCFs). β-catenin/Lef/TCF combinations are capable of modulating the expression of an extensive array of target genes. Prominent osteogenic and chondrogenic factors, including Runx2 and Ihh, are included among these [82]. Furthermore, Wnt/β-catenin signaling has the ability to modulate the transcription of key factors involved in bone homeostasis and remodeling, RANKL and OPG [78, 83]. The activity of the Wnt/β-catenin signaling pathway can be hindered by various skeletogenic factors and can also be regulated by transmembrane inhibitors (Kremen1/2, Ror2, and Ryk) and secreted Wnt antagonists (Dkks, Sfrps, Wif1, and Sost) [84]. The transition from epithelial-to-mesenchymal of the cranial neural crest cells, as well as their breakdown and translocation into distinctive cranial regions, are all dependent on canonical Wnt signaling [85].

The regulatory crosstalk between the Wnt and EDA pathways is among the most studied EDA crosstalk in the skeletal system and it influences various aspects of bone, cartilage, and tooth development, homeostasis and morphogenesis in the tooth and craniofacial or dermal skeletal tissues of model fish and mammals. During tooth development, EDA is known to enhance the expression of WNT10A and WNT10B via NF-κB signaling, promoting the differentiation of odontoblasts and ameloblasts [65]. Similarly, the interaction between these pathways is essential for craniofacial skeletal patterning, with evidence showing that Wnt signaling acts upstream of EDA during bone formation, although it inhibits osteoblast differentiation [33, 86]. This upstream regulatory effect is also observed in palatogenesis, where Pax9-induced upregulation of Wnt signaling via suppression of DKK, an inhibitor of Wnt, leads to the induction of EDA signaling and bone formation [87]. In zebrafish, the cooperation between Wnt and Eda/NF-κB signaling facilitates a signaling wave essential for scale bone morphogenesis [73, 88]. This cooperative relationship is also significant in cichlid and stickleback teleosts, where Wnt/Lef1 signaling is suggested as an upstream regulator of EDA during the morphogenesis of dermal bones such as scales and armor plates [41, 43], suggesting a teleost counterpart to Wnt–EDA interactions described in mouse and human tooth and craniofacial tissues. These findings provide evolutionary parallels to Wnt–EDA cooperation in dermal skeletal patterning, and complement mammalian data in which Wnt signaling modulates EDA activity during tooth and craniofacial bone development, even though the exact regulatory architecture differs between dermal and endochondral contexts.

In studies related to endochondral skeletal tissues, the interaction between EDA and Wnt signaling may occur indirectly through EDA’s major downstream effectors, NF-κB and NFATc1. For instance, NF-κB can induce *RSPO2* expression during inflammation, activating Wnt signaling and subsequently inhibiting cartilage formation, while also participating in a negative feedback loop that may be essential for late-stage chondrogenesis [89–91]. In this negative feedback loop, RSPO2 mediated activation of Wnt signal can later block Nf-kb activity. It is important to note that the Nf-kb mediated induction of RSPO2 can be triggered by both inflammation and mechanical overload [89]. The Wnt pathway activity is required for the late stage of chondrocyte differentiation (chondrocyte hypertrophy) while Nf-kb induces chondrocyte apoptosis; therefore this negative feedback might be required for promoting the late stage of chondrocyte differentiation without entering apoptosis [90, 91]. Moreover, Wnt signaling plays a crucial role as a major inhibitor of NFATc1, therefore controlling osteoclast differentiation. This inhibition is mediated through both canonical (GSK3β/β-catenin) and non-canonical (WNT4, WNT16, WNT5A, WNT3A) pathways, highlighting the multifaceted nature of Wnt signaling in bone resorption and remodeling [92–95]. Taken together, these findings highlight the complex and integral interactions between the Wnt and EDA pathways in craniofacial skeletal tissues, and raise the possibility that similar relationships may operate in endochondral skeletal tissues, as well (Fig. 1C). At the physiological level, their interactions may be essential for skeletal tissue homeostasis, growth, and remodeling.

### Notch Signaling Pathway

Notch proteins are transmembrane receptors that are exceptionally conserved. They comprise three domains: extracellular, transmembrane, and intracellular [96]. By interacting with the extracellular domain of Notch, the canonical ligands Jagged and delta-like (Dll) facilitate the unloading of the intracellular domain. When inducing the expression of target genes, including Hes and Hey, the liberated domain assembles with its transcriptional regulator CSL (RBPjk) [96]. A multitude of biological processes, especially those involving the determination of cell fate, are remarkably governed by the canonical Notch signal, which is an incredibly simple molecular cascade. In addition to early somitogenesis, skeletal growth, and bone remodeling, the pathway is implicated in various facets of skeletal development [97–99]. Chondrocyte and osteoblast differentiation are initially inhibited by Notch signaling, which subsequently initiates chondrogenesis [100]. By differentially regulating genes such as RANKL and OPG, the pathway also influences osteoclastogenesis throughout lineage commitment and maturation [97, 98, 101]. Notch signaling hinders the differentiation of chondrocytes as well as osteoblasts at various phases [98, 102].

The regulatory crosstalk between the Notch and EDA pathways has been found to play significant role in certain processes involving the development and differentiation of skeletal tissues across various species. For instance, a recent study in stickleback fish has shown that mutations in *Eda* along with alterations in Notch signaling components, specifically *Dld* and *Egfl6*, impact the development of lateral plates by influencing osteoblast differentiation in these structures [103]. Similarly, in pufferfish, the development of dermal spines, a scale derivative, is regulated by the interaction between Eda signaling and Notch3 expression [104]. These teleost studies point to evolutionary parallels in which Eda–Notch interactions regulate dermal osteoblast differentiation, whereas in mouse bone marrow, the interaction between Notch signaling and EDA pathways manifests differently; for example, the overexpression of *Jagged1* and *Notch2* enhances the transcription of *NFATc1*, promoting osteoclast differentiation [105]. Conversely, in mouse jaw bone, suppression of Notch3 leads to reduced expression of its downstream targets (*Jag1* and *Hey1*), which in turn increases *NFATc1* expression and thereby promotes osteoclast differentiation [106].

In endochondrally derived skeletal tissues, the EDA-Notch regulatory connection extends to the inhibition of osteoblast differentiation where Notch1 and their signaling through RBPjk are found to suppress osteoblast activity by inhibiting NFATc1 [107, 108]. Furthermore, under inflammatory conditions in bone, the opposing roles of RBPjk and NFATc1 regulate miR-182, which plays a stimulatory role in osteoclast differentiation and inflammatory bone resorption [109]. In cartilage, activation of Notch1/2 signaling leads to the suppression of NFATc1 and inhibition of early-stage chondrocyte differentiation, illustrating a divergent role in cartilage compared to bone [110]. Collectively, these studies highlight the complex interactions between Notch and EDA pathways in skeletal development, emphasizing their pivotal roles in regulating cell differentiation and tissue formation (Fig. 1D). Hence, EDA-Notch interactions may play a crucial physiological role in regulating skeletal homeostasis by modulating osteoblast and osteoclast differentiation, potentially contributing to balanced bone formation and resorption in both endochondral and craniofacial skeletal tissues.

## Cross-Talk Between EDA and Growth Factor-mediated Signals

In contrast to the core developmental pathways discussed above (BMP, Hedgehog, Wnt, Notch), the evidence for direct cross-talk between EDA and growth factor–mediated signals such as FGF, IGF and MAPKs in skeletal tissues is still fragmentary and often indirect. In many cases, regulatory connections are inferred because both EDA and these pathways converge on common transcriptional hubs (for example, NF-κB or NFATc1), rather than being demonstrated by direct biochemical interaction assays in bone or cartilage cells. The subsections below should therefore be read primarily as hypothesis-generating syntheses that highlight plausible routes of integration between EDA and growth factor signals, rather than as comprehensive lists of experimentally validated mechanisms.

### Fibroblast Growth Factor Signaling Pathway

A large family of primarily paracrine ligands known as fibroblast growth factors (FGFs) activates numerous conserved signaling pathways. FGF signaling is fundamental at various phases of vertebrate development and is involved in a vast array of biological processes [111]. FGFs generate signals via FGF receptors (FGFRS), which comprise a family of tyrosine kinases. Due to alternative splicing that is strictly regulated, the quantity of FGFR isoforms in vertebrates significantly surpasses the quantity of genes that encode them [112]. During development, FGFs and FGFR isoforms exhibit discrete spatiotemporal expression patterns, and their function disruption has been linked to an assortment of developmental and morphological abnormalities [113, 114]. Various aspects of endochondral and intramembranous bone development, chondrogenesis, and bone mechanical sensing are regulated by FGF-regulated pathways [113, 115, 116].

**Fig. 2:**
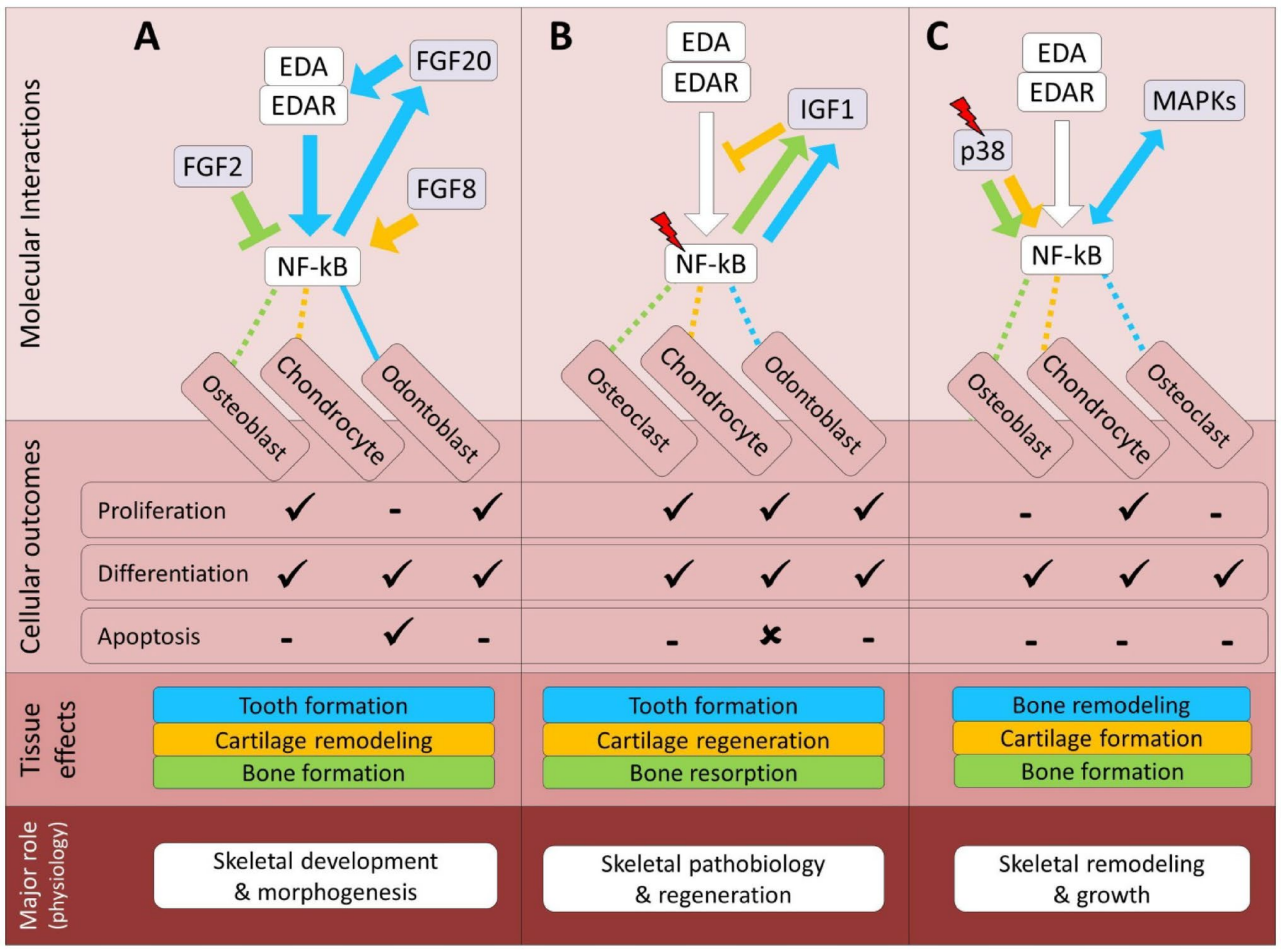
Predicted regulatory crosstalk of the EDA pathway components with growth factors mediated signals during skeletogenesis. (**A–C**) Molecular and cellular details of EDA signaling crosstalk with the FGF, IGF, and MAPK pathways, respectively, along with their associated skeletal outcomes at the tissue and physiological levels. In the molecular interactions section, arrows and block heads represent regulatory induction and inhibition, respectively. The color of lines connecting EDA signal to each cell type follows the color of the specified interaction, and dashed and solid lines respectively represent indirect and direct regulatory connections between the upstream components of EDA and other pathways. In addition, red slash symbols above certain genes indicate upstream components that detect mechanical signals within each molecular signaling. The color coding of skeletal effects corresponds to the colors of related regulatory interactions in the molecular section. In the cellular section, ticks and crosses denote the promotion and impairment of processes, based on the crosstalk studied. The lowest section presents the primary expected physiological role of EDA in the context of its crosstalk with each pathway

FGF signaling pathway is an immediate target of EDA during tooth development and *Fgf20* is an essential downstream effector of Eda and affects Eda-regulated characteristics of tooth morphogenesis, including the number, size and shape of teeth [117]. Studies in ectodermal tissues also proposed *Fgf20* as a key mediator and a direct target of EDA signaling transduction [118, 119], and interestingly, the absence of Fgf20 also leads to the suppression of *EDAR* expression and, consequently, also EDA signaling [6]. These findings suggest a synergistic and reciprocal crosstalk between EDA and FGF signals at transcriptional levels in these tissues. In zebrafish, activation of both EDA and FGF signals are essential for osteoblastogenesis during scale development, however, their direct interaction in this process remained unexplored [120]. In cichlid fish, a positive expression correlation between *eda* and *fgf20* has been shown during morphogenesis of posterior scales [43]. These fish data thus provide functional analogies for EDA–FGF cooperation in dermal skeletal morphogenesis that complement the more direct Eda–Fgf20 relationship described in mammalian tooth development and the indirect FGF–NF-κB–EDA interactions implicated in mammalian bone and cartilage. An indirect interaction between EDA and FGF signals is indicated during zebrafish scale development through the Wnt signaling pathway [73], thus their crosstalk in scale osteoblasts might not be the result of a direct transcriptional activation. On the contrary, a study of endochondrally-driven rat and mouse osteoblasts has shown that activation of FGF signaling through FGF2 can also suppress NF-kB signaling and promote bone formation [121]. Compared to bone and tooth, less is known about potential crosstalk between EDA and FGF signals in cartilage, although a recent study in endochondral mammalian chondrocytes has found that FGF8 induces the expression of ECM components through induction of NF-κB transcriptional activity [122]. Taken together, these observations suggest the presence of direct crosstalk in the tooth and indirect crosstalk in bone and cartilage between EDA and FGF signals in the vertebrate models examined so far (Fig. 2A). The interactions appear to be synergistic and reciprocal, with broader implications for the physiological role of their coordination in skeletal development and morphogenesis in these systems.

### Insulin-Like Growth Factor Signaling Pathway

Insulin-like growth factors (IGFs), initially identified in the musculoskeletal system, mediate growth and differentiation [123]. They activate an evolutionarily conserved signaling cascade involving IGFs, IGF receptors, IGF binding proteins (IGFBPs), and IGFBP proteases. IGFs bind to activated receptors, initiating gene regulatory signals via the MAPK and PI3K-AKT pathways. IGFBPs, regulating IGF bioavailability, impact various facets of IGF function. Present in all tissues, IGFs play crucial roles in homeostasis, embryonic/postnatal development, adaptive morphogenesis and tissue survival [124–127]. Predominant in bones, IGFs promote mineralization, differentiation, and formation but exert a multifaceted impact on bone [128]. IGF-mediated signaling regulates chondrocyte proliferation, differentiation, and apoptosis in mouse and rat cartilage models [129].

During follicular hair formation, EDA acts upstream of IGF signaling indirectly through Shh-dependent transcriptional regulation of IGFBP-5, a major IGF inhibitor in various tissues [130]. Moreover, IGFBP-3 can modulate EDA transcription through a Wnt/β-catenin-dependent mechanism during tooth mineralization development [131]. To date, no study has been conducted to investigate direct crosstalk between EDA and IGF signals in any skeletal cells, even though direct regulatory interactions between NF-kB and IGF-1 mediated signals have been found in both bone and cartilage under normal and pathological conditions [132–134]. During cartilage inflammation, IGF-1 directly inhibits NF-kB transcriptional activity, leading to promotion of chondrogenesis and cartilage repair [133]. During endochondral ossification in the mouse growth plate, the promoting effects of NF-kB transcriptional activity are mediated through activation of IGF-1 signaling pathway [132]. Also, during mechanical stress in the endochondral growth plate, the biomechanical signals induce NF-κB transcriptional activation and IGF-1 signal appears to act downstream of NF-κB signal in this condition; however, the detailed regulatory connection has not been studied [135]. Moreover, NF-κB signal-dependent bone resorption deficiency affects tooth root development from failure of releasing of IGF-1 from bone matrix through osteoclasts and IGF-1 inhibition in root odontoblasts [136]. These results suggest that NF-κB signal can act upstream of the IGF-1 signaling pathway and activate it in both osteoclasts and odontoblasts. Taken together, these observations indicate various crosstalk between EDA and IGF signals in skeletal cells in different vertebrate models. The regulatory nature of these can be very different, as IGF-1 inhibits NF-κB in regenerating cartilage, whereas NF-κB signal stimulates IGF-1 activity during bone and cartilage growth, tooth root development, cartilage mechanical overloading and bone resorption (Fig. 2B). A broader physiological aspect of their interaction may be their coordinated role in skeletal pathobiological processes in the specific experimental and clinical contexts studied so far.

### Signals Mediated by MAPKs

The conserved family of serine/threonine kinases, mitogen-activated protein kinases (MAPKs), plays a crucial role in transducing external signals into cells via membrane receptors [137]. Major regulatory cascades, including extracellular signal-regulated kinase (ERK), c-Jun NH2-terminal kinase (JNK), and p38 MAPK, are incorporated within MAPKs. Despite growth factors being primary activators, each MAPK cascade mediates unique cellular signals related to apoptosis, stress, differentiation, and growth [138–140]. Ap-1 complex members (c-Jun and c-Fos heterodimer) regulate gene expression during osteoblast differentiation and are targeted by distinct MAPKs. Crucially, MAPK cascade activation is involved in mesodermal derivatives, skeleton, and dentition formation. Bone mechanotransduction is facilitated through Ap-1 transcriptional activity induction via JNK and ERK [141].

The essential role of p38 MAPK signaling pathway during tooth morphogenesis and enamel secretion has been shown to be independent of EDA signaling [142]. During ectodermal differentiation, however, Edar mediated signal has been found to directly (but modestly) activate MAPK/JNK [143]. Though no study has ever investigated presence of direct crosstalk between MAPK and EDA signaling pathways, it is well known that activated NF-κB acts as potent inhibitor of JNK by which cell survival versus cell death in various tissues may be balanced [144]. Conversely, activated MAPK pathway (including JNK signal) enhances osteoclast differentiation through induction of NF-κB transcriptional activity in human bone cells [145]. Interestingly, a recent study has shown that activated NF-κB can also induce osteoclast differentiation through activation of MAPK signal components (ERK, JNK and p38) in mouse osteoclast precursors [146]. These suggest that a synergistic activation of NF-κB and MAPK signals is required for bone remodeling and both signals can act upstream of each other during this process. In endochondral-type chondrocytes, p38 MAPK signaling acts directly at upstream of NF-κB, and enhanced p38 activity induces NF-κB signal, leading to promotion of both chondrocyte differentiation and inflammation [147]. Similarly, mechanical pressure in bone activates p38 MAPK signaling and in turn enhanced p38 activity again induces NF-κB signal leading to osteogenesis osteoblast differentiation [148]. Taken together, these studies indicate potential crosstalk between MAPK and EDA signaling pathways in various skeletogenic processes, which is most likely mediated through synergistic activation of NF-κB dependent signals (Fig. 2C). At a broader physiological level, their interaction might be a potential future research topic in processes involving skeletal remodeling under normal or pathological conditions.

## Cross-Talk Between EDA and Signals Mediated by Nuclear Receptors

Compared with the canonical morphogen pathways, mechanistic data on direct cross-talk between EDA and nuclear receptor–mediated signals in skeletal cells are still limited. Much of the discussion in this section is based on (i) studies in non-skeletal tissues, and (ii) the shared use of NF-κB and NFATc1 as downstream effectors, from which we infer potential routes of interaction. The relationships outlined here should therefore be mainly viewed as working models and hypotheses that may guide future experiments, rather than as firmly established EDA-dependent mechanisms in bone and cartilage.

### Retinoic Acid Signaling Pathway

Retinoic Acid (RA), among the initial vertebrate morphogens [149], plays diverse roles in developmental patterning. Its inactive precursor, Vitamin A (retinol), is vital for growth, development, and tissue maintenance [150, 151]. RA rapidly diffuses and activates specific heterodimeric nuclear receptors, primarily RXRα/RAR (α, β, and γ), influencing RA-responsive gene expression through distinct sequences. In vertebrates, RA signaling coordinates early skeletal morphogenesis and anterior-posterior patterning during embryogenesis by regulating homeobox gene expression [149, 152]. Dysregulation of RA synthesis and signaling, including variations in RA receptor-encoding genes and enzyme-encoding genes (Rdh10 and Raldh3), can induce various skeletal abnormalities [153–155]. RA-metabolizing enzymes, linked to skeletal development, impact spatiotemporal RA levels post-synthesis.

**Fig. 3:**
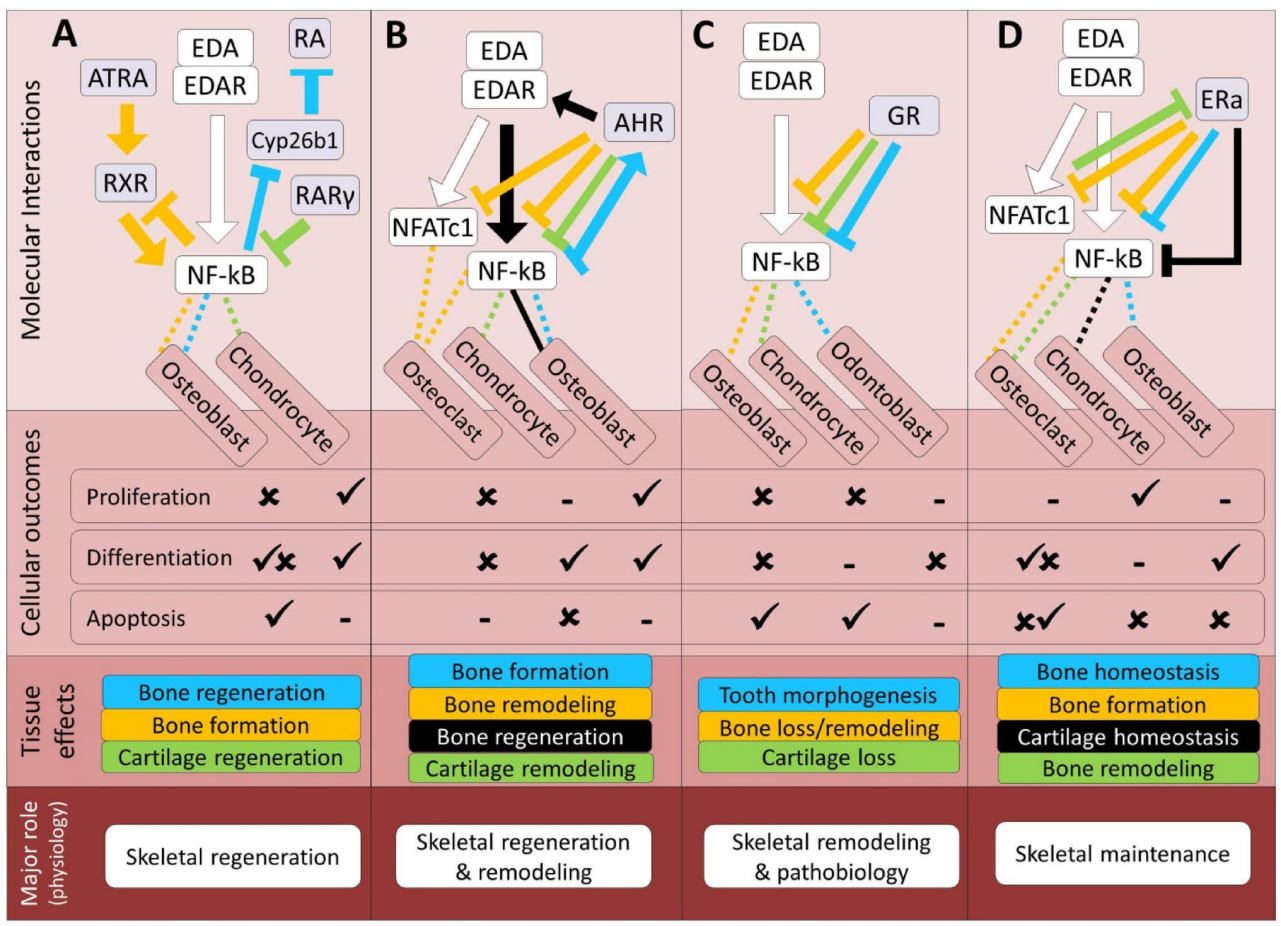
Predicted regulatory crosstalk of the EDA pathway components with nuclear receptor signals during skeletogenesis. (**A–D**) Molecular and cellular details of EDA signaling crosstalk with the RA, AHR, GR and estrogen pathways, respectively, along with their associated skeletal outcomes at the tissue and physiological levels. In the molecular interactions section, arrows and block heads represent regulatory induction and inhibition, respectively. The color of lines connecting EDA signal to each cell type follows the color of the specified interaction, and dashed and solid lines respectively represent indirect and direct regulatory connections between the upstream components of EDA and other pathways. The color coding of skeletal effects corresponds to the colors of related regulatory interactions in the molecular section. In the cellular section, ticks and crosses denote the promotion and impairment of processes, based on the crosstalk studied. The lowest section presents the primary expected physiological role of EDA in the context of its crosstalk with each pathway

Coordinated activation of both RA and EDA signals are proposed to be essential for tooth development and morphogenesis in multiple vertebrate systems examined so far; however, the detailed molecular interactions between the two pathways have remained unexplored during this process [156]. In several metabolic diseases, RXRα has been found to bind to EDA promoter and induced EDA transcription [6]. In human skin, EDAR has been found to be induced by activation of RA signaling pathway [157]. Such a direct transcriptional regulation of EDA or EDAR by RA pathway has not been reported in skeletal cells. However, during endochondral skeletogenesis, RA mediated suppression of NF-κB transcriptional activity is proposed as a molecular mechanism underlying chondrogenic effects of RA during cartilage regeneration [158]. On the contrary, all-trans-retinoic acid (ATRA), an active vitamin A compound binding to RXRα/RAR and activating their regulatory function, inhibits osteogenesis by enhancing NF-κB transcriptional activity in human periodontal ligament [159]. Strikingly, a recent study in adult zebrafish has demonstrated that NF-κB signaling acts upstream of RA signaling during osteoblast dedifferentiation which is essential for skeletal regeneration [160]. This is done through suppression of the RA-degrading enzyme cyp26b1 by activated NF-κB signaling. This zebrafish mechanism provides a functional analogy for how NF-κB–RA interactions might contribute to bone repair in mammals, although a direct EDA-dependent counterpart in mammalian fracture healing has not yet been demonstrated. Previously, in mammals, it has been shown that NF-κB impair osteoblastogenesis through inhibition of VDR and RXR function [161]. These observations indicate potential direct and indirect reciprocal crosstalk between RA and EDA signals, which may involve both inhibitory and stimulatory effects, during skeletogenesis (Fig. 3A). Furthermore, their interaction may represent a promising avenue for future research into the mechanisms underlying skeletal regeneration at a broader physiological level.

### Aryl Hydrocarbon Signaling Pathway

The Aryl hydrocarbon/Dioxin receptor (Ahr), a member of the bHLH-PAS family of heterodimeric TFs, initially identified for mediating a signaling pathway, is implicated in various skeletogenic phases [162]. At developmental level, for instance, Ahr loss-of-function mutation can cause craniofacial and skeletal phenotypes with elongation along the anterior-posterior axis [163–166]. Activated Ahr pathway may influence skeleton formation via various skeletogenic factors, dependent on time, dose, and ligand. The direct regulatory link between Ahr and EDA pathways in the skeletal system is only reported in zebrafish during craniofacial and fin skeletal development [167]. This connection is proposed to be through Ahr2 and Edar, by which Ahr2 seems to act at upstream of *Edar* inducing its expression during skeletal development [167]. Another likely scenario can be EDA regulation through interaction between NF-κB and cyp1a1, the main target of Ahr pathway [167], via competition with NF-κB in transcriptional regulation of cyp1a1 [168]. In mammals, Ahr mediated transcriptional induction of *Edar* is only demonstrated in human and rodent liver cells [169]. Thus, the Ahr2–Edar regulation described in zebrafish currently represents an evolutionary and mechanistic parallel that has not yet been confirmed in mammalian skeletal tissues, and may also point to lineage-specific differences in how Ahr and EDA signaling intersect during craniofacial development.

During inflammatory responses in different tissues, it is already known that NF-κB induces *Ahr* expression by directly binding to the Ahr promoter [170]. Moreover, the Ahr pathway modulates osteoclast differentiation and bone remodeling through regulation of NF-κB nuclear translocation or competing with its transcriptional activity in osteoclasts [168]. Interestingly, the Ahr pathway can also enhance osteoclastogenesis through crosstalk with NF-κB signal [168]. The differentiation of osteoblasts is also hindered by the Ahr pathway through a complex mechanism involving NF-κB signal modulation [170, 171]. In cartilage tissue, activation of Ahr pathway by its endogenous ligands can reduce inflammation in chondrocytes by blocking NF-κB signal [172]. These findings implicate both inhibitory and stimulatory regulatory connections between the Ahr pathway and NF-κB mediated signals in various aspects of skeletogenesis (Fig. 3B). Although the molecular mechanisms underlying potential direct crosstalk between Ahr and EDA signaling pathways in skeletal cells remain poorly understood, existing findings raise the possibility that their interaction may be important for coordinating skeletal remodeling and regeneration at a broader physiological level.

### Glucocorticoid Signaling Pathway

Derived from steroids, glucocorticoids (GCs) bind to glucocorticoid receptors (GR) present in virtually all tissues [173]. GCs traverse cell membranes, modulating transcription via nuclear GR post-conversion to an active state. Ligand-bound GR regulates gene transcription positively or negatively by interacting with other TFs. GR-mediated signaling is implicated in various aspects of skeletogenesis and morphological adaptation of skeletal structures [174, 175]. GC-induced osteoporosis results from GR pathway interactions with signals regulating skeletal cell processes [175]. Essential GR signaling elements respond to environmental and cellular stresses [176]. Maternal GR transcripts in zebrafish embryos are crucial for early skeletal development [177]. Elevated GC levels during growth can subtly manifest craniofacial and vertebral skeletal abnormalities in zebrafish larvae and in mammalian models such as rabbits and humans [178–181]. Major genes involved in ECM biogenesis, including ctsk, dcn, and mmp2/9/13, are among the direct downstream effectors of the GR pathway during skeletogenesis [179, 182]. Differential regulation of these genes during growth results in distinctive morphological modifications to skeletal structures.

Multiple components of GR and EDA signaling pathways are required for hair, skin and tooth development and morphogenesis [183, 184]. In mice, the effects of induced activation of the GR pathway (through *GR* overexpression) is mediated by reduction of NF-κB transcriptional activity and interference with NF-κB function, which is the result of significant decrease in NF-κB binding activity in tooth epithelium [184]. The same study also suggests that the GR signaling pathway can interfere with the NF-κB function at multiple levels during tooth development [184]. In mice, GR signaling can inhibit normal osteoblastogenesis in the absence of NF-κB activity indicating independency of GR signal during this process [185]. However, under inflammatory conditions, GR signaling appears to interfere NF-κB mediated signals in bone via hindering NF-κB transcriptional activity or directly regulating its downstream target genes [186]. Similarly, in articular cartilage, GR signaling has been found as a strong suppressor of NF-κB transcriptional activity and function in cartilage under inflammatory condition [187]. These findings indicate inhibitory effects of GR-dependent signaling on the EDA pathway (through suppression of NF-κB activity) during normal tooth development, with a potentially broad physiological role in pathological conditions affecting bone and cartilage (Fig. 3C).

### Estrogen Signaling Pathway

Oestrogens, often known as estrogens, are hormones derived from precursor molecules with androgenic properties. While initially identified as sex hormones, they have the ability to impact a range of developmental and physiological processes, such as the creation and regeneration of the skeletal system [188, 189]. Given the prevalence of sexual dimorphism, which is ultimately influenced by sex-hormone signaling, this is not unexpected. In skeletal cells, oestrogens transmit signals through two types of receptors: ER-alpha/-beta, which are estrogen receptors regulated by ligands [188], and receptors connected with G-proteins (e.g., GPR-30 and GPER1) [190–192]. The mediators of estrogen signals are present in chondrocytes and are involved in the process of chondrogenesis [192, 193]. The impact of estrogen on the multiplication of chondrocytes and the development of cartilage varies among different species [194, 195]. High levels of estrogen during zebrafish development can completely interfere with the construction of craniofacial and trunk skeletal structures [195, 196].

In zebrafish, the expression of *edar* and *esr2a* (estrogen receptor 2a) are both up-regulated in similar areas of the epidermis at initiator sites of ectodermal/dermal appendage where developing scales appear [197]. In mammalian epithelial cells, selective activation of estrogen receptor-β (*Er-a*) induces *Edar* transcription [198]. These observations provide an evolutionary parallel suggesting that estrogen receptors can modulate EDA/EDAR expression in both teleost and mammalian ectodermal tissues. Nonetheless, there has been no investigation into the potential direct crosstalk between EDA and estrogen signaling pathways in skeletal tissues, and it remains unclear whether similar regulatory links exist in mammalian bone and cartilage. This is particularly surprising because estrogen signaling has been well known as a potent repressor of NF-κB activity in various skeletal cells such as osteoblasts, osteoclasts and chondrocytes [199–201]. Estrogen signaling can also inhibit osteoclast differentiation through transcriptional repression of *NFATc1*, the master regulator of osteoclastogenesis [202]. Furthermore, NFATc1 has been found to promote osteoblast differentiation by suppressing *Er-a* transcription [203]. In this study, NFATc1 was found to act as an upstream transcriptional inhibitor of the *Er-a* gene by directly binding to its promoter [203]. However, a recent study has found unexpectedly that a direct cooperative interaction between Er-a and NFATc1 leads to suppression of *WNT5B* transcription and consequently to promotion of osteoblast differentiation in human [204]. Based on these observations, EDA and estrogen signaling pathways might have indirect regulatory interactions in skeletal tissues through competitive modulation of NF-κB and NFATc1 activities; such crosstalk of EDA and estrogen signaling pathways would be more likely to have inhibitory outcomes (Fig. 3D). At a physiological systems level, the interaction between EDA and estrogen signaling may contribute to maintaining skeletal tissue homeostasis and should be explored in future studies.

## Cross-Talk Between EDA and Calcium Dependent Pathways

### Signaling Pathways Mediated by Nuclear Factor of Activated T-Cells

The NFAT (nuclear factor of activated T-cells) signaling pathway plays a role in various aspects of skeletal development and morphogenesis, orchestrating processes such as osteoblastogenesis, osteoclastogenesis as well as skeletal remodeling, inflammation and homeostasis [205–207]. This pathway, which is induced by calcium through the Orai1 calcium channel and STIM1 calcium sensor, is involved in transducing extracellular signals into intracellular responses, ultimately regulating gene expression and cellular functions essential for skeletal development. NFAT pathway activation promotes the differentiation of mesenchymal stem cells into osteoblasts [208]. Studies have shown that NFATc1, a key transcription factor downstream of calcineurin, plays a central role in this process by regulating the expression of osteogenic genes such as Runx2 and Osterix [208]. The NFAT pathway also plays a crucial role in regulating the differentiation and function of osteoclasts [209]. NFATc1 is a master regulator of osteoclastogenesis, and its activation promotes osteoclast differentiation by inducing the expression of genes essential for osteoclast formation and activity, including TRAP and Cathepsin K [209]. Moreover, NFATc1 regulates the expression of genes involved in osteoclast fusion and bone resorption, thereby contributing to bone remodeling [205]. NFATc1 regulates the balance between osteoblast and osteoclast activities, thereby modulating bone remodeling processes such as bone formation, resorption, and turnover. Dysregulation of NFATc1 activity can disrupt this balance, leading to pathological conditions such as osteoporosis and osteopetrosis [205, 210]. Interestingly, the conservation of the NFAT pathway between mammals and fish makes it an interesting target for comparative molecular studies of bone remodeling across vertebrates [211]. Given that NFATc1 is also a major downstream effector of EDA/NF-κB signaling in skeletal cells, this conservation supports the idea that EDA–NFAT modules identified in one vertebrate group (for example, in teleost models) may often represent functional analogies, and in some cases genuine evolutionary parallels, to EDA–NFAT-driven processes in mammalian bone remodeling. Moreover, the NFAT pathway is involved in regulating bone and cartilage inflammation. NFATc1 activation in immune cells, such as macrophages and T cells, promotes the production of pro-inflammatory cytokines and mediators, which can contribute to bone and cartilage inflammation [209]. Finally, NFAT pathway plays a critical role in maintaining skeletal homeostasis by regulating the expression of genes involved in bone and cartilage metabolism, mineralization, and turnover [212]. NFATc1 activation modulates the activity of osteoblasts and osteoclasts in response to various extracellular signals, thereby ensuring proper skeletal development, growth, and maintenance throughout life [213].

**Fig. 4:**
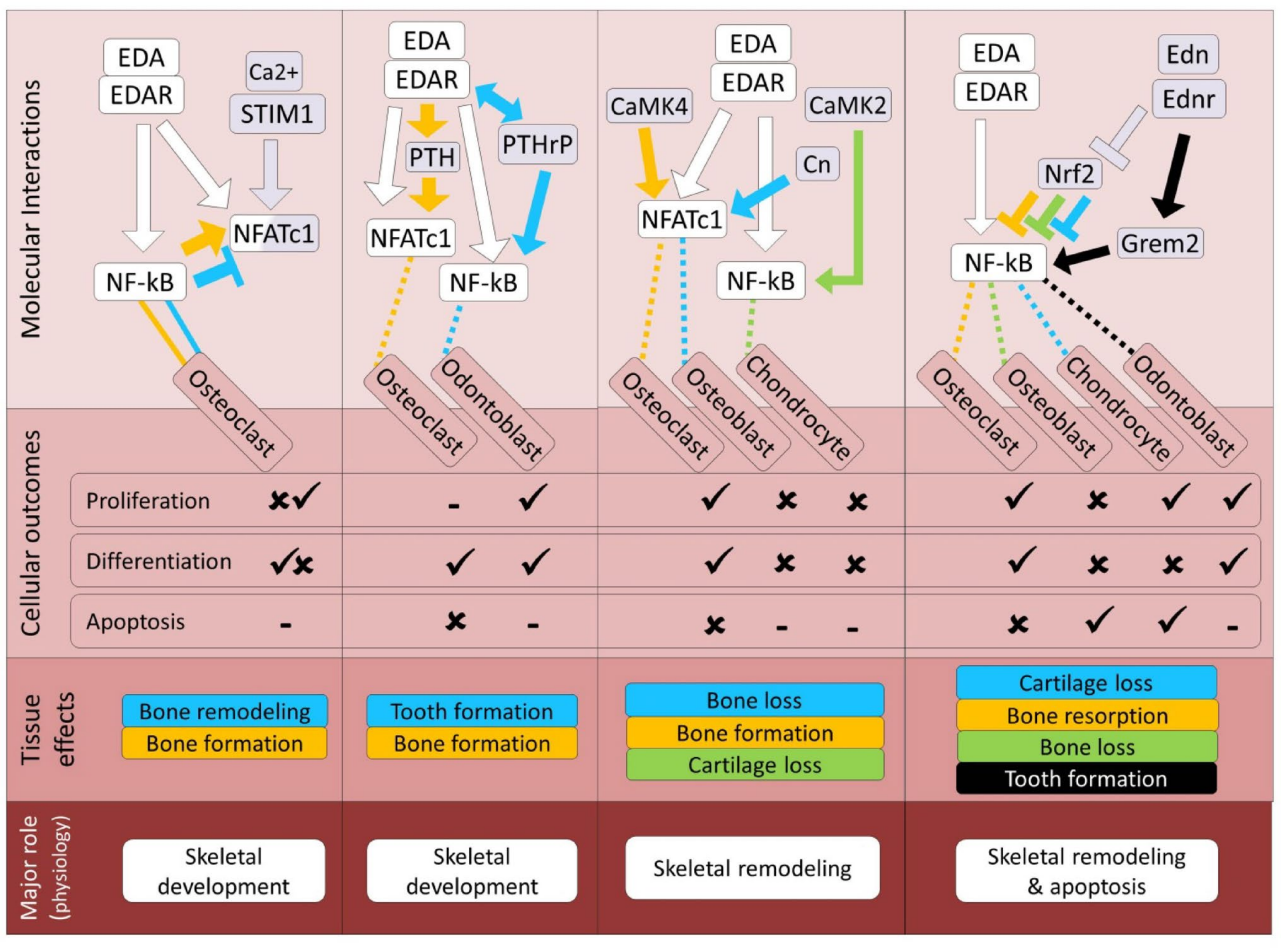
Predicted regulatory crosstalk of the EDA pathway components with signals mediated by calcium-dependent pathways during skeletogenesis. (**A–D**) Molecular and cellular details of EDA signaling crosstalk with the NFAT, PTH/PTHrP, Ca²⁺/CaM and Edn/Ednr pathways, respectively, along with their associated skeletal outcomes at the tissue and physiological levels. In the molecular interactions section, arrows and block heads represent regulatory induction and inhibition, respectively. The color of lines connecting EDA signal to each cell type follows the color of the specified interaction, and dashed lines represent indirect regulatory connections between the upstream components of EDA and other pathways. The color coding of skeletal effects corresponds to the colors of related regulatory interactions in the molecular section. In the cellular section, ticks and crosses denote the promotion and impairment of processes, based on the crosstalk studied. The lowest section presents the primary expected physiological role of EDA in the context of its crosstalk with each pathway

Among the calcium-dependent signaling pathways, EDA signal has the most direct and well-characterized crosstalk with the NFAT pathway [27, 214]. This is due to the fact that *NFATc1* (the major transcription factor in the NFAT pathway) is also one of the most prominent downstream transcriptional targets (after *NF-κB*) of the EDA signaling pathway in skeletal tissues and EDA treatment can potently induce transcription of both *NFATc1* and *NF-κB* in both craniofacial and endochondral skeletal cells [27, 214]. However, a recent study in mice has shown that enhancement of NF-κB signal can impair osteoclastogenesis through direct inhibition of NFATc1 activity as well [215]. Previously, NF-κB was always considered as a potent transcriptional inducer of *NFATc1* during osteoclast differentiation [213]. This contrasting evidence may indicate the presence of an unknown negative feedback loop in which NF-κB signal limits NFATc1 activity in skeletal cells, but further functional studies are required to validate this hypothesis. Although the possibility for the presence of such a feedback is proposed in osteoclasts under inflammatory condition, the potential involvement of EDA signal in this remained elusive [216]. Considering the extensive role of NFAT pathway in various aspects of skeletogenesis (Fig. 4A), it is plausible that future studies might reveal additional contexts in which EDA signal interferes with these processes, through modulation of NFAT pathway, although current evidence directly links EDA to only a subset of NFAT-dependent phenomena.

### Parathyroid Hormone Signaling Pathway

Parathyroid hormone (PTH) and parathyroid hormone-related peptide (PTHrP) are closely related proteins secreted by distinct cell types. PTH, from parathyroid glands, regulates calcium and phosphate levels in the bloodstream. PTHrP, with RNA-splicing variations, plays critical roles in growth and maturation as paracrine/autocrine hormones [217]. They can attach to different or overlapping receptors, triggering diverse signaling pathways, including elevated Ca2+, activation of enzymes like PKA and PLC, and modulation of pathways like MAPK [217]. The PTH/PTHrP pathways regulate osteoblastogenesis and bone formation [218, 219]. PTHrP signaling influences Sox9 and Runx2, key proteins for cartilage and bone formation and controls RANKL and Ap-1 activity in skeletal cells.

In mammals, it has been already shown that *PTHrP* is a direct downstream transcriptional target of Eda/Edar/NF-κB in epithelial cells in skin and mammary glands and its expression is induced by EDA treatment during mammary gland morphogenesis [220]. Interestingly, the impairment in bone remodeling and osteoclast differentiation in HED patients with EDA mutation is attributed to reduced PTH function since the expression of PTH is decreased in their craniofacial skeletal tissues [33]. This indicates that EDA signal may act upstream of the PTH pathway during skeletogenesis; however the molecular mechanisms underlying such a regulatory connection have not been further explored. Because PTH has crosstalk with Wnt and FGF pathways in skeletal tissues, it is also likely that the regulatory connection between EDA and PTH signals is indirect and mediated through these pathways. Such a scenario is worth investigating since Eda/Edar/NF-κB pathway targets both *PTHrP* and *Wnt10* in epithelial cells where they exhibit similar expression pattern [221]. Notably, cooperative regulatory interactions between EDA, PTHrP, Wnt and FGF signaling pathways have been already demonstrated during tooth formation in mammals [222]. Taken together, these findings imply on presence of a stimulatory regulatory connection between EDA and PTH/PTHrP pathways in skeletal tissues which in the vertebrate models studied so far, might be indirect through Wnt and FGF pathways (Fig. 4B). Although limited data are available to interpret the roles of EDA and PTH/PTHrP pathways in the general physiology of the skeletal system, mechanistically their interaction is a plausible contributor to key skeletal developmental processes in these systems.

### Calmodulin Signaling Pathway

Calcium (Ca2+) is a ubiquitous signaling molecule regulating Ca2+-binding factors and associated cascades. It plays a vital role in cellular processes and is integral to skeletal biology [223, 224]. Stimulated cells experience a rapid Ca2 + release through voltage-sensitive channels, increasing cytoplasmic Ca2 + that binds to calmodulin (CaM), a conserved protein. CaM, binding calcium ions, activates proteins like CaM kinases (CaMK) and Calcineurin (Cn), which are crucial for skeleton formation. Ca2+/CaM signals regulate bone processes, influencing osteoblast and osteoclast differentiation and proliferation [224, 225]. They also affect chondrocyte differentiation, mechanotransduction signals, and interact with MAPKs, CREB, NFAT. The Ca2+/CaM signal interacts with the BMP pathway, influencing skeletal development [223, 226, 227]. Differentially regulated components of the Ca2+/CaM pathway contribute to skeletal variation in closely related species [226, 227].

Similar to EDA signal, the pathway mediated by Ca2+/CaM is known to be essential during tooth development and morphogenesis [228] and recently it has been shown that mutations in components of both pathway can result in similar dental deformities in human [229]. Nevertheless, there is no study investigating the possibility of direct crosstalk between EDA and Ca2+/CaM signaling pathways. However, it is demonstrated that during endochondral ossification, a CaM kinase (CaMKII) can inhibit chondrogenic differentiation of progenitor cells through activation of the two major downstream transcription factor targets of EDA signal (NF-κB and NFATc1) [230]. On the other hand, activation of another CaM kinase (CaMKIV) has been found to induce osteoclast differentiation again through enhancement of NF-kB and NFATc1 activity in mouse and human osteoclast precursor cultures [231]. Calcineurin (Cn) is another major target protein that is activated by Ca2+/CaM signal, and strikingly, activation of Cn inhibits osteoblast proliferation and differentiation through direct dephosphorylation of NFATc1 in mouse and rat bone cells [224]. Despite the absence of demonstrated direct crosstalk between EDA and Ca2+/CaM signaling pathways, the regulation of key targets of the EDA signal by components of the Ca2+/CaM signal in skeletal cells (Fig. 4C) warrants further investigation into potential direct interactions during skeletogenesis. Overall, based on the findings discussed above, it is conceivable that interactions between EDA and Ca²⁺/CaM could become a future topic in skeletal physiology, particularly in processes related to skeletal remodeling.

### Endothelin Signaling Pathway

Endothelins (Edns), initially produced as inert proteins, undergo complex enzymatic processes and are secreted by cells in response to stimulation [232]. Edns act in both paracrine and autocrine manners, binding to transmembrane receptors (Ednrs) that initiate downstream signaling cascades, increasing Ca2 + levels and activating MAPK and PI3K-AKT pathways. The endothelin signaling pathway is a critical regulator in skeletal biology, influencing both bone and cartilage development [233]. Endothelins, a family of peptides, interact with endothelin receptors to modulate cellular processes such as proliferation, differentiation, and matrix production. In bone, endothelin signaling supports osteoblast activity and bone matrix deposition, contributing to skeletal growth and remodeling. In cartilage, it plays a role in chondrocyte proliferation and the maintenance of cartilage structure, essential for joint function and integrity [234]. Dysregulation of endothelin signaling is associated with skeletal abnormalities and disorders such as osteoarthritis, emphasizing its importance in maintaining healthy bone and cartilage dynamics.

In the skin of EDA-deficient mice, *Edn1* has been found to be a transcriptional target of EDA signaling and *Edn1* expression is reduced in the absence of active EDA signal in keratinocytes [235]. Although, EDA and Edn/Ednr signaling pathways have been shown to be essential for jaw skeletogenesis and tooth development [236], surprisingly, to date no study has investigated their potential regulatory interaction during skeletal development and morphogenesis. A major target of Edn/Ednr during craniofacial skeletal development is *Nr2f*, and activation of Edn signal strongly suppresses Nr2f transcription in developing skeleton of mouse and zebrafish upper jaw derivatives [237]. *Nr2f* encodes a crucial transcription factor regulating various aspects of bone and cartilage development and remodeling [238]. Strikingly, Nr2f is a very potent suppressor of *NF-κB* transcription in skeletal cells, particularly during osteoclastogenesis and bone remodeling [238]. Therefore, it is conceivable that the activated Edn/Ednr signal could enhance the effects of the EDA/NF-κB signal in bone by repressing *Nr2f* transcription, but confirmation of such an indirect regulatory synergy between these pathways in skeletal tissues requires experimental validation. Finally, the activation of both EDA and Edn/Ednr/Grem2 signaling pathways are essential during tooth development, indicating their potential cooperative/synergistic interactions in skeletal development [239]. Based on these findings, the interaction between EDA and Edn/Ednr may play an important role in skeletal tissue remodeling, particularly in processes involving skeletal cell apoptosis in the tissues and vertebrate models studied (Fig. 4D).

## Potential Cross-talk Between EDA and Non-canonical Microenvironment-responsive Pathways Affecting Skeletogenesis

The potential interactions between EDA signaling and microenvironment-responsive pathways, such as serotonin, integrins and nitric oxide, are even less directly characterized than those described above. Here, we mainly synthesize indirect evidence: EDA’s downstream effectors (particularly NF-κB and NFATc1) are known to intersect functionally with these pathways in skeletal cells, and in some cases EDA and these signals are co-active in the same tissues. Accordingly, the cross-talk scenarios proposed in this section should be understood as speculative but biologically motivated hypotheses, highlighting possible mechanisms by which EDA could be influenced by, or contribute to, mechanical and metabolic cues in skeletal tissues.

### Serotonin Signaling Pathway

The serotonin signaling pathway plays a multifaceted role in skeletal development and maintenance, acting both locally within bone and cartilage and systemically through its endocrine effects [240]. Serotonin, a neurotransmitter, exists in two distinct pools: central (produced in the brain) and peripheral (produced in the gut), with the latter being particularly influential in bone biology. Peripheral serotonin can inhibit bone formation by acting on osteoblasts, while central serotonin has been shown to promote bone mass accrual through neural signaling pathways. In cartilage, serotonin regulates chondrocyte proliferation and matrix production, contributing to proper cartilage formation and maintenance [241, 242].

**Fig. 5:**
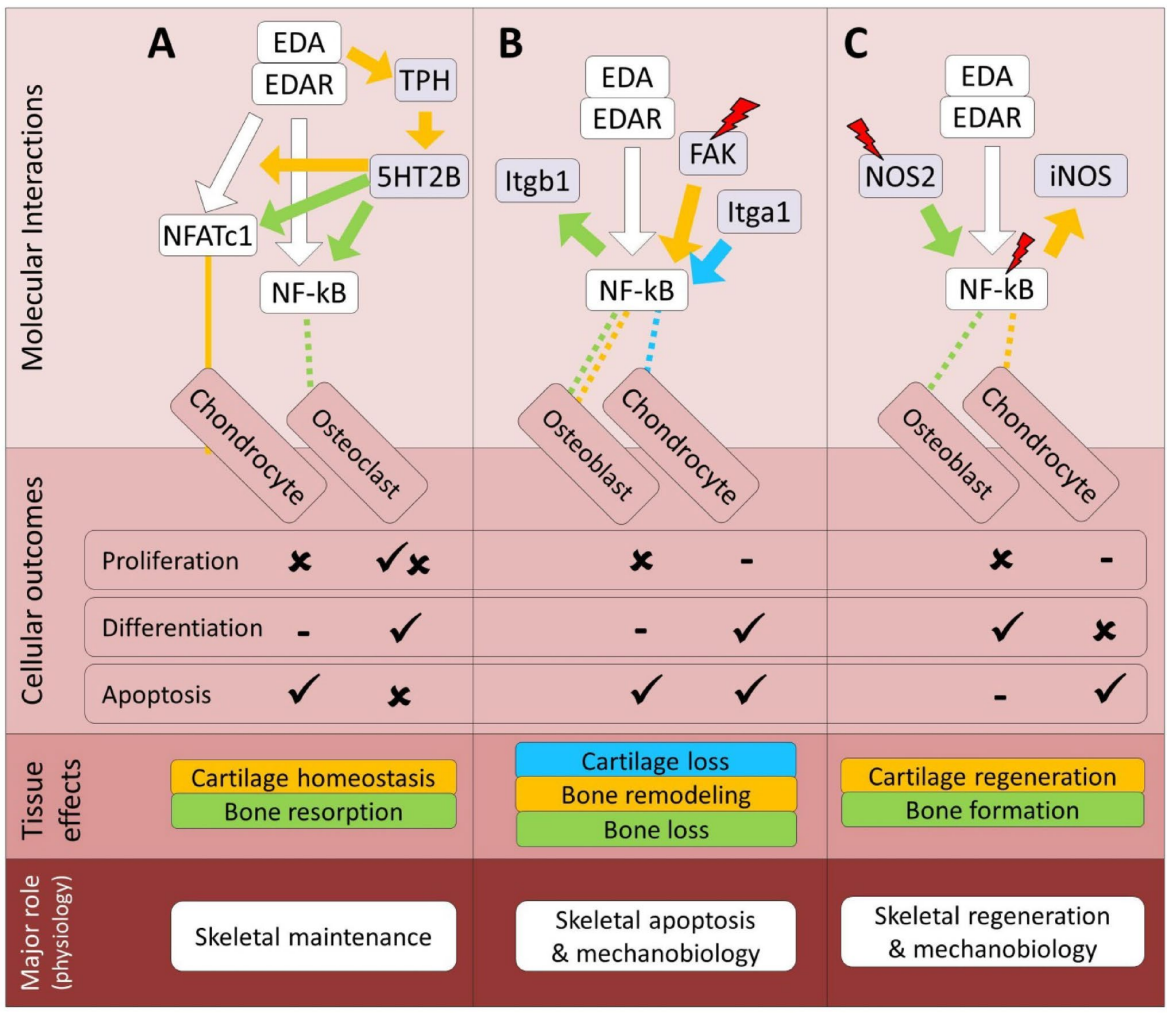
Predicted regulatory crosstalk of the EDA pathway components with non-canonical microenvironment-responsive pathways. (**A–C**) Molecular and cellular details of EDA signaling crosstalk with the serotonin, integrin and nitric oxide pathways, respectively, along with their associated skeletal outcomes at the tissue and physiological levels. In the molecular interactions section, arrows and block heads represent regulatory induction and inhibition, respectively. The color of lines connecting EDA signal to each cell type follows the color of the specified interaction, and dashed lines represent indirect regulatory connections between the upstream components of EDA and other pathways. Additionally, red slash symbols above certain genes indicate upstream components that detect mechanical signals within each molecular signaling. The color coding of skeletal effects corresponds to the colors of related regulatory interactions in the molecular section. In the cellular section, ticks and crosses denote the promotion and impairment of processes, based on the crosstalk studied. The lowest section presents the primary expected physiological role of EDA in the context of its crosstalk with each pathway

In zebrafish, the development of skin and scales both involve activation of serotonin and EDA signaling pathways, but the potential molecular crosstalk between these pathways has not been investigated [243]. These observations in teleost dermal tissues therefore provide a suggestive functional analogy to the situation in mammalian bone, where serotonin and EDA-related NF-κB/NFATc1 signaling both influence osteoclastogenesis, but a direct molecular link between the two has not yet been established. Currently, no research has explored the potential direct molecular interaction between serotonin and EDA signaling pathways in skeletal tissues. However, studies of EDA-deficient mice indicated that EDA mediated regulation of osteoclast differentiation is tightly linked to activation of RANKL (Eda1/Edar/NF-κB axis) [27], a major factor controlling osteoclastogenesis, and interestingly, in endochondral bone, serotonin signal also exerts its stimulatory effects on osteoclastogenesis through activation of NF-κB and NFATc1 [244]. This may indicate synergistic effects of serotonin and EDA signaling pathways on bone resorption and remodeling through NF-κB and NFATc1. Moreover, the adverse effects of excessive activation of 5-HT2B receptor on endochondral bone in mouse models and in clinical observations in humans appeared to be linked with transcriptional dysregulation of *NF-κB*, the main transcription factor in EDA signaling [245]. These also suggest a potential competitive regulatory effect of serotonin and EDA signaling pathways on *NF-κB* transcription in skeletal cells. In addition, NF-κB function is implicated in tryptophan metabolism, which is required for serotonin synthesis, and reciprocally, serotonin signal mediates its effects on inflammatory responses through regulation of *NF-κB* transcription [246]. Finally, a complex indirect regulatory link between activity of EDA signaling and expression of tryptophan hydroxylase (*TPH*), encoding the main enzyme in serotonin synthesis, has been reported in cartilage [247]. Taken together, these findings suggest potential indirect synergistic (through RANKL transcription and tryptophan metabolism) or competitive (through NF-κB transcription) regulatory connections between serotonin and EDA signaling pathways which requires further validations in skeletal tissues (Fig. 5A). From a systemic physiological perspective, their interactions may be important for advancing our understanding of skeletal tissue maintenance.

### Integrin Signaling Pathway

Integrins are transmembrane receptors that facilitate ECM interactions, playing a pivotal role in skeletal development and maintenance. In bone biology, integrins such as α1β1 and α2β1 mediate osteoblast adhesion to collagen, influencing bone formation and remodeling processes [248, 249]. In cartilage, integrins like α5β1 and αVβ3 are expressed on chondrocytes and interact with ECM components, regulating cell adhesion, mechanotransduction, and matrix production [250]. These integrin-mediated interactions are essential for maintaining cartilage integrity and function. Dysregulation of integrin signaling has been implicated in skeletal disorders, including osteoarthritis, where altered integrin expression contributes to disease progression.

There have been no investigations examining the possible direct molecular interplay between integrin and EDA signaling pathways in skeletal tissue. In epithelial cells, however, EDA signaling pathway modulates the affinity of adhesion receptors such as integrins, thus affecting integrin-mediated cell-matrix morphogenesis in these cells [251]. In the skin of EDA-deficient mice, the direct physical interactions between the extracellular domain of EDA and matrix elements like integrins are lost suggesting that the EDA mediated skin morphogenesis is exerted through direct physical interactions with integrin molecules [252]. A network-based analysis of molecular players during scale development and morphogenesis also revealed extensive regulatory connections between integrins and EDA signaling components [253]. Similarly, potential regulatory connections between the activity of EDA and integrin-mediated signals have been reported in human cartilage, providing a mammalian counterpart to the teleost scale data. These observations suggest that EDA–integrin interactions in teleost dermal scales and mammalian cartilage are likely to represent functional analogies, and possibly evolutionary parallels, in which EDA signaling modulates or responds to integrin-mediated cell–matrix communication in mechanically active skeletal tissues. It is important to note that NF-kB signaling directly modulate integrin-β1 expression in bone and acts upstream of integrin signaling [254]. On the other hand, activation of certain integrin signals can activate NF-kB mediated signals in osteoblasts in endochondral bone upon mechanical stimulation [255], but the potential involvement of EDA in this regulatory connection has remained unclear. Given these observations, it is conceivable to propose a hypothetical model in which EDA activates integrin signaling through direct physical interaction with integrin molecules during skeletogenesis (Fig. 5B), emphasizing the need for further investigations to explore this possibility. Moreover, based on the current cellular findings, the interaction between EDA and integrin signaling may have a broader physiological impact on skeletal mechanobiology and apoptosis-related processes, although this remains to be directly tested in vivo.

### Nitric Oxide Signaling Pathway

Initially, nitric oxide (NO) signaling was discovered to regulate endochondral ossification and later was found to be involved in skeletal cell differentiation and mechanical adaptation [256–258]. To date, there is no study investigating the possibility of direct molecular crosstalk between NO and EDA signaling pathways in any skeletal tissue; however, NF-κB, the main transcription factor mediating EDA signal, has been shown to directly act upstream of the nitric oxide synthase II encoding gene (*NOS2*) and increases its transcription in the brain [259]. Moreover, in several ectodermal-derived tissues, NF-κB activates the transcription of iNOS, another major gene encoding inducible nitric oxide synthase [260]. In articular cartilage of mammalian joints, NF-κB activates the transcription of iNOS in chondrocytes in response to inflammatory stress [261]. A later study in endochondral cartilage has shown that the mechanotransduction signal mediated by NF-κB in cartilage might involve activation of NOS signal in addition to other mechanosensing signals in mammalian models [262]. Furthermore, the inhibition of NF-κB signal is essential during endochondral cartilage repair and this mechanism appears to also involve activation of NO signaling [263]; however, further studies are required to elucidate the molecular interaction between NF-κB and NO signals in cartilage regeneration/repair. In endochondral bone, NO signaling (through NOS2) is required for mediating the mechanical-induced responses and inhibition of NO signaling reduces osteoblast proliferation and increases their differentiation [264]. Interestingly, this mechanism in bone is also mediated by NF-κB at the transcriptional level [264]. These findings indicate the necessity of future research to explore potential direct molecular crosstalk between components of EDA and NO signaling pathways in skeletal system since they both share the same transcription factor (NF-κB) in these processes (Fig. 5B). Furthermore, their interactions may prove to be relevant in broader physiological contexts, particularly in studies of mechanical sensing and tissue regeneration within skeletal systems.

## Conclusion and Conceptual Framework

The Ectodysplasin-A (EDA) signaling pathway is frequently studied in the context of dermal skeletal development, particularly in craniofacial bone and cartilage, where it can significantly influences the formation and diversity of skeletal structures in the vertebrate models studied so far, especially in fish and mammals. Its interactions with other critical skeletogenic and morphogenic pathways that we synthesize in this review highlight the incredibly complex network of molecular cross-talk governing skeletal development and morphogenesis (see summary Table 1). In interpreting this table, it is important to note that interactions classified with D denote experimentally demonstrated EDA–pathway links, whereas entries that include only In1 and/or In2 summarize indirect, NFATc1- or NF-κB-based inferences and should therefore be viewed as provisional models for future testing. By integrating signals from multiple pathways, EDA may contribute to the precise regulation of cellular processes essential for various aspects of skeletal physiology (see summary Table 1) – making it a coordinator of sculptors. At the same time, the available data still come from a relatively limited set of experimental systems, and many proposed interactions remain indirect or hypothetical, particularly in endochondral bone and cartilage.

**Table 1:** Summary of the experimentally demonstrated and hypothesized crosstalk between EDA and skeletogenic signaling pathways

Pathway	Bone	Cartilage	Tooth	References
TGF-β	Unknown	Unknown	Antagonistic (D, In2)	[20, 24, 42–44]
BMP	Synergistic (D, In1)	Unknown	Antagonistic (D, In1)	[19–21, 52–54]
Hh	Both (D)	Synergistic (D)	Synergistic (D)	[2, 20, 21, 33, 52, 62–65]
Wnt	Both (D, In1, In2)	Both (D, In1, In2)	Synergistic (D, In1, In2)	[23, 31, 33, 54, 62, 73–82]
Notch	Both (In1, In2)	Antagonistic (In1, In2)	Unknown	[90–97]
FGF	Synergistic (D, In2)	Synergistic (D, In2)	Synergistic (D, In2)	[6, 33, 62, 107–112]
IGF	Both (In1, In2)	Both (In1, In2)	Both (In1, In2)	[119–125]
MAPK	Synergistic (In2)	Synergistic (In2)	Unknown	[131–137]
RA	Both (In2)	Both (In2)	Synergistic (In2)	[6, 144–149]
Ahr	Both (In2)	Both (In2)	Unknown	[153–158]
GR	Antagonistic (In2)	Antagonistic (In2)	Antagonistic (In2)	[169–173]
ER	Antagonistic (In1, In2)	Antagonistic (In1, In2)	Unknown	[185–192]
NFAT	Both (D, In1, In2)	Both (D, In1, In2)	Synergistic (D, In1, In2)	[17, 201–204]
PTH/PTHrP	Synergistic (D, In2)	Synergistic (D, In2)	Synergistic (D, In2)	[23, 208–210]
Ca2+/CaM	Both (In1, In2)	Antagonistic (In1, In2)	Unknown	[212, 216–219]
Edn/Ednr	Synergistic (D, In2)	Both (D, In2)	Synergistic (D, In2)	[223–227]
Serotonin	Both (In1, In2)	Both (In1, In2)	Unknown	[17, 231–235]
Integrin	Both (D, In2)	Both (D, In2)	Unknown	[239–243]
NO	Synergistic (In2)	Both (In2)	Unknown	[244–252]

This table summarizes the nature of molecular crosstalk between Ectodysplasin-A (EDA) signaling and major skeletogenic signaling pathways in bone, cartilage, and tooth tissues. Each cell indicates the overall interaction type between EDA and the respective pathway as interpreted from studies discussed in this article: ‘Synergistic’, ‘Antagonistic’, ‘Both’ (if dual effects are reported), or ‘Unknown’ (if no relevant data exists). The type of regulatory link is provided in parentheses: D = direct interaction (through EDA or EDAR); In1 = indirect interaction via NFATc1; In2 = indirect interaction via NF-κB. Multiple terms (e.g., D, In1, In2) reflect convergence of multiple regulatory inputs. Entries that include D reflect cases where direct EDA–pathway interactions have been demonstrated in skeletal contexts, whereas entries containing only In1 and/or In2 are based on indirect evidence through shared downstream effectors and should be regarded as provisional, hypothesis-generating relationships rather than established mechanistic interactions in skeletal cells

Viewed from a systems perspective, EDA is therefore best regarded not as a universal master regulator, but as a context-dependent “conductor” whose influence depends on how it engages a small set of shared signaling hubs and downstream transcription factors. Among these, NF-κB and NFATc1 emerge as central integrators through which EDA can interface with a wide range of skeletogenic and microenvironment-responsive pathways, including BMP, Hedgehog, Wnt, FGF, TGF-β, RA, Ahr, GR, estrogen, IGF, MAPKs, PTH/PTHrP, Ca²⁺/CaM, endothelin, serotonin, integrins and nitric oxide.

Within this framework, at least three broad “layers” of EDA-mediated orchestration can be distinguished. First, at the level of direct ligand–receptor interactions, EDA and its receptors (EDAR/XEDAR) participate in relatively well-defined cross-talk with pathways such as BMP, Hedgehog, Wnt and FGF, for example by modulating ligand expression or being regulated by these pathways in return. Second, at the level of transcriptional integration, EDA-induced NF-κB and NFATc1 activity provides a shared node through which growth factor, nuclear receptor and calcium-dependent signals are filtered and combined, thereby influencing osteoblast, osteoclast and chondrocyte behaviour even when no direct biochemical interaction with EDA or EDAR has been demonstrated. Third, at the microenvironmental level, mechanically and hormonally responsive pathways (such as integrin, NO, serotonin, GR and estrogen signaling) can tune the sensitivity and output of EDA–NF-κB–NFATc1 modules, adding an additional layer of context dependence during skeletal remodeling and regeneration.

The relative contribution of these layers is likely to differ across skeletal compartments and vertebrate lineages. In teleost dermal elements (for example, scales and fin rays), current evidence points to a prominent role for direct EDA cross-talk with Wnt, BMP and Hedgehog signals during patterning and growth of epidermis-associated skeletal tissues. In contrast, in mammalian craniofacial and endochondral bones, EDA appears to influence bone-cell biology more indirectly, by modulating NF-κB- and NFATc1-dependent transcriptional programs that intersect with PTH/PTHrP, IGF, MAPK, nuclear receptor and Ca²⁺/CaM pathways. Thus, the same EDA-centred core module can orchestrate very different skeletogenic outcomes, depending on which upstream pathways feed into NF-κB/NFATc1 and which downstream targets are available in a given cell type and developmental stage.

In light of the evidence reviewed here, several concrete questions emerge. How context-specific is EDA’s “conductor” role across dermal versus endochondral skeletal elements and across fish and mammals? To what extent do NF-κB and NFATc1 act as shared hubs through which EDA prioritizes or filters inputs from BMP, Wnt, Hedgehog, growth factors and nuclear receptors? And how do mechanical, hormonal and microenvironmental signals modulate EDA-dependent outcomes during remodeling and regeneration? Addressing these questions will require combining comparative approaches in fish and mammals with targeted genetic and cell-biological experiments in defined cell types [265]. Understanding these complex relationships enhances our comprehension of skeletal biology and highlights potential avenues for therapeutic intervention in skeletal disorders, such as craniofacial skeletal anomalies in humans. Future research focusing on EDA’s multifaceted roles and its synergistic actions with other signaling networks holds in these defined contexts promise for advancing skeletal biology and regenerative medicine.
